# Use of a new genetic element to improve expression level of cell lines for recombinant protein production

**DOI:** 10.1186/1753-6561-9-S9-P4

**Published:** 2015-12-14

**Authors:** Emilie Vaxelaire, Lauriane Gamand, Christel Aebischer-Gumy, Pierre Moretti, Martin Bertschinger

**Affiliations:** 1 Glenmark Pharmaceutical SA, 2300 La Chaux de Fonds, Switzerland

## Background

Naturally occurring chromatin modifying elements such as MAR, UCOE or cHS4 were identified from the genome of higher eukaryotes. It is well documented that the presence of these regulatory elements leads to better recruitment of transcriptional machinery and/or prevents epigenetic silencing mechanisms [1,2]. The goal of this work was the evaluation of a new genetic element to improve transgene expression, from the flanking sequences of the glyceraldehyde 3-phosphate dehydrogenase (GAPDH) gene, which is a ubiquitously expressed enzyme. It was hypothesized that the surrounding genetic environment of the gene may lead to a DNA structure favorable for transgene expression in eukaryotes. In this study, we have investigated the effect on stable and transient expression in CHO and HEK293 cells of the 3.2 kilo base pairs (kb) sequences flanking upstream (5') and downstream (3') the GAPDH locus.

## Materials and methods

For vector development, three kb were amplified by PCR from the 5' and 3' sequences flanking the human GAPDH gene. The upstream GAPDH sequence cloned in 5' and the downstream GAPDH sequence was cloned in 3' of the expression cassette. The vectors containing the 5' and 3' GAPDH flanking regions were called "GAPDH plasmids". The GAPDH plasmids were transfected into suspension-adapted CHO and HEK293 EBNA cells. The effect of the upstream and downstream sequences were observed in transiently and stably transfected cells. Two different expression plasmids (GAPDH-A and GAPDH-B) were tested in comparison to original backbone (pGLEX41). "GAPDH-A" corresponds to pGLEX41 modified by the addition of the GAPDH flanking regions, the codon optimization of the bla gene and the pUC origin of replication which was replaced by the R6K origin of replication. "GAPDH-B" is the same as "GAPDH-A" with an additional reduction in the number of CpGs in certain regions of the backbone. The expression level of transient and stable transfections was studied by analyzing the IgG titer in the supernatant. To assess the effect of GAPDH flanking regions on stability in CHO pools stably transfected, intracellular staining was performed to quantify the percentage of cells expressing both LC and HC among each stable pool. In addition, methylation of the promoter was detected using the bisulfite conversion and sequencing method.

## Results

Table 1 shows the transient expression level of an IgG in CHO and HEK293 EBNA cells. A significant increase in transient expression was obtained with the GAPDH plasmids in both host cell lines for both constructs. Data demonstrated that the beneficial effect of the vector is solely due to the GAPDH flanking sequences and not the A and B modification (codon and CpG modification, data not shown). Compared to the original vector backbone (pGLEX41), a 2.7 to 3-fold higher expression could be observed in CHO cells. In HEK293 EBNA cells, the GAPDH-B vector is showing a 3-fold, whereas the GAPDH-A vector shows an even higher increase in expression (5-fold) compared to the pGLEX41 vector. In transient, the GAPDH flanking regions is favorable for the production of IgG in both host cell lines used.

**Table 1:** Transient expression of IgG antibody in HEK293 EBNA and CHO cells.

IgG titer average in transient expression (μg/ml)	Cell lines	pGLEX41	GAPDH-A	GAPDH-B
	HEK293 EBNA (N = 3, +/- STDEV)	16.1 (+/-1.35)	86.6 (+/- 2.15)	53.9 (+/- 2)
	CHO (N = 2, +/- MinMax)	2.07 (+/- 0.13)	5.74 (+/- 0.69)	6.33 (+/- 0.02)

IgG titers measured respectively on day 10 and 5 post-transfection.

**Figure 1:**
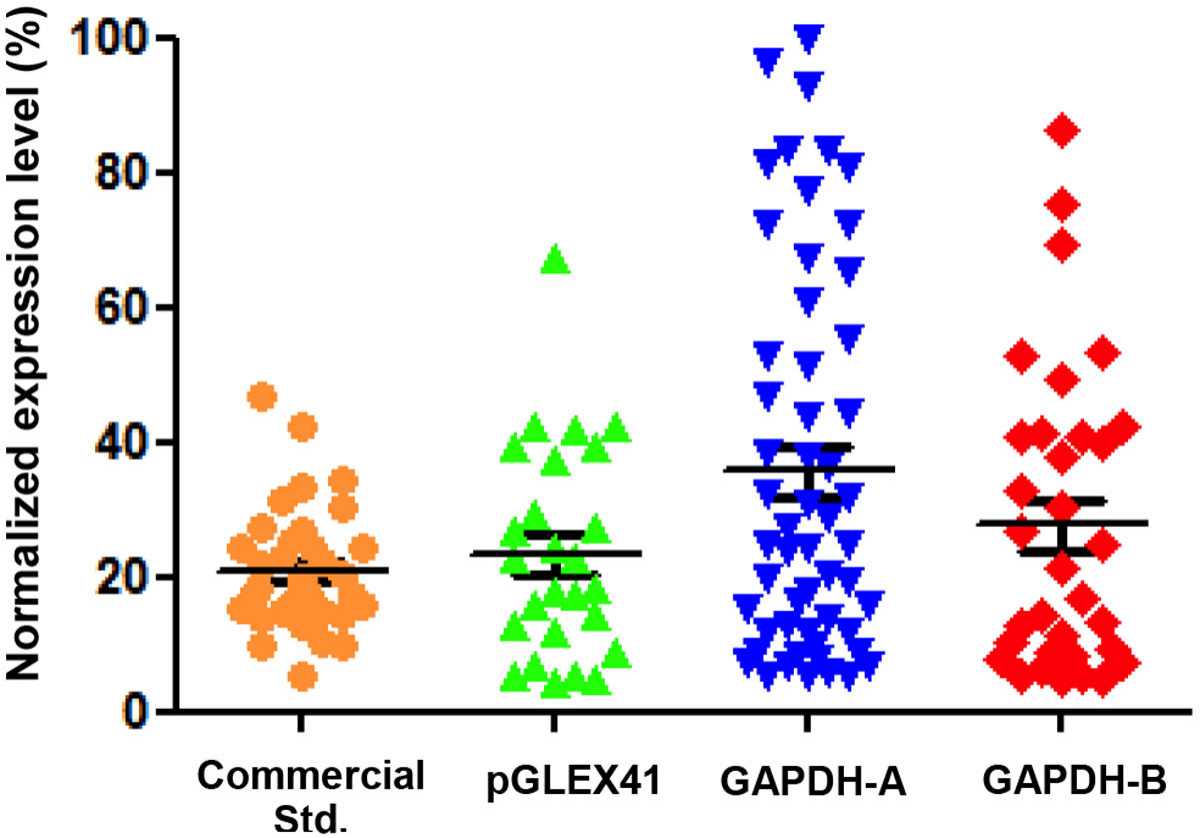
**Normalized expression level of IgG after a 6 days supplemented batch of stable CHO pools in 96 deep well plate format (Mean, 26 < N < 55)**. Normalization has been performed on the highest titer obtained among the four conditions. The original backbone pGLEX41 has been used as a control.

The positive effect of the GAPDH flaking regions observed in transient set-up was verified in stable pools. Figure 1 shows the expression level of stable CHO pools generated with a standard containing a commercial available insulator (commercial Std.), pGLEX41, GAPDH-A and GAPDH-B vectors. Transfections performed with GAPDH-A induced a higher IgG expression than pGLEX41 and the commercial standard transfections. In addition, the "GAPDH vectors" increase the number of high expressing pools. Therefore, the beneficial effect of the GAPDH flanking regions is not only valid for episomal expression but also after integration in the CHO genome.

The impact of the GAPDH flanking regions on stability was assessed on stable pools using intracellular staining for light chain (LC) and heavy chain (HC). The experiment was done on CHO pools transfected with pGLEX41, GAPDH-A and GAPDH-B plasmids and a plasmid containing a commercial available insulator (Median, 49 < N < 118). Results from intracellular content showed that the commercial insulator worked better for supporting stability with a median value higher than 95 %, whereas the best condition is obtained with GAPDH-A with a median around 60% (data not shown). Methylation at the promoter level was measured over generations on stable pools containing the GAPDH vectors. Methylation of the mCMV promoter was evidenced in clones harboring the GAPDH vectors. The GAPDH vectors did not show a positive impact on epigenetic silencing in this experimental set-up, in particular on promoter methylation. Nevertheless, stable clonal cell lines with superior expression levels can be generated with these vectors. Stability was assessed for different mAb over 60 generations with expression titers up to 3 g/L without further process optimization.

## Conclusions

The implementation of human genomic sequences flanking the GAPDH gene in expression vectors leads to a 2-5 fold increase in protein expression in CHO and HEK. The effect on expression is most likely due to a - previously unknown - enhancer activity of the GAPDH flanking regions [3]. However, the elements did not demonstrate a "barrier" effect preventing the spread of methylation and heterochromatin formation after transgene integration. Nevertheless, stable CHO cell lines can be easily isolated and demonstrated superior expression levels compared to control vectors.
